# Metformin exerts anti-AR-negative prostate cancer activity via AMPK/autophagy signaling pathway

**DOI:** 10.1186/s12935-021-02043-2

**Published:** 2021-08-17

**Authors:** Chunyang Chen, He Wang, Xinyu Geng, Dongze Zhang, Zhengyu Zhu, Guangbo Zhang, Jianquan Hou

**Affiliations:** 1 Department of Urology, Wuxi People’s Hospital Affiliated to Nanjing Medical University, 299 Qingyang Road, Wuxi, 214023 Jiangsu People’s Republic of China; 2 Department of Urology, The First Affiliated Hospital of Soochow University, 188 Shizi Street, Suzhou, 215006 Jiangsu People’s Republic of China; 3 Jiangsu Institute of Clinical Immunology, The First Affiliated Hospital of Soochow University, 708 Renmin Road, Suzhou, 215006 Jiangsu People’s Republic of China; 4 Jiangsu Key Laboratory of Clinical Immunology, Soochow University, 708 Renmin Road, Suzhou, 215006 Jiangsu People’s Republic of China; 5 Department of Urology, Dushu Lake Hospital Affiliated to Soochow University, 9 Chongwen Road, Suzhou, 215006 Jiangsu People’s Republic of China

**Keywords:** Metformin, Autophagy, Prostate cancer, AMPK, LC3B

## Abstract

**Background:**

Encouraged by the goal of developing an effective treatment strategy for prostate cancer, this study explored the mechanism involved in metformin-mediated inhibition of AR-negative prostate cancer.

**Methods:**

Cell behaviors of DU145 and PC3 cells were determined by CCK8 test, colony formation experiment and scratch test. Flow cytometry was used to detect cell cycle distribution. Cell autophagy was induced with metformin, and an autophagy inhibitor, 3-MA, was used to assess the level of autophagy. Detection of LC3B by immunofluorescence was conducted to determine autophagy level. Cell proliferation, autophagy and cell cycle were examined by performing Western blot. DU145 and PC3 cell lines were transfected with AMPK siRNA targeting AMPK-α1 and AMPK-α2. Tumor formation experiment was carried out to evaluate the anti-prostate cancer effect of metformin in vivo.

**Results:**

The inhibitory effect of metformin on the proliferation of prostate cancer cell lines was confirmed in this study, and the mechanism of such an effect was related to autophagy and the block of cell cycle at G0/G1 phase. Metformin also induced the activation of AMPK, markedly promoted expression of LC3II, and down-regulated the expression of p62/SQSTM1. Animal experiments showed that the tumor volume of metformin group was smaller, meanwhile, the levels of p-AMPK (Thr172) and LC3B were up-regulated and the Ki-67 level was down-regulated, without abnormalities in biochemical indicators.

**Conclusion:**

This study found that autophagy induction might be the mechanism through which metformin suppressed the growth of AR-negative prostate cancer. Moreover, the activation of AMPK/autophagy pathway might be a therapeutically effective for treating AR-negative prostate cancer in the future.

## Background

Prostate cancer is main cause of cancer deaths among men in the world, despite great improvement in its therapies in the past decades [1, 2]. A majority of patients are diagnosed with prostate cancer at an advanced stage, thus missing the optimal opportunity for better treatment. Prostate cancer with metastasis into bone requires to be treated by the combination of androgen deprivation, chemotherapy, and radiation. However, in castrate-resistant prostate cancer (CRPC), bone-metastatic outgrowth is difficult to be prevented, directly or indirectly leading to an unfavorable long-term survival [3]. Therefore, effective treatments should be developed for a better cancer management.

Metformin is a commonly used clinical drug to lower blood glucose for patients with type 2 diabetes mellitus. Recent studies have shown that metformin also has anti-tumor effects in vitro and in vivo [4, 5]. A number of systematically conducted observational studies demonstrated that metformin treatment could reduce the risk of developing cancer and related mortality as well as improving the prognosis of patients with cancer and diabetes [6–8]. Moreover, the improvement of metformin to the prognosis of prostate cancer has also been previously reported [9]. Though study found that metformin could prevent tumorigenesis of prostate cancer, whether metformin is also effective to prostate cancer patients without diabetes remains to be investigated [10]. The effectiveness of metformin in preventing colorectal cancer, ductal carcinoma in situ oral tumor, squamous cell carcinoma, endometrial cancer has been confirmed in the past [11–14], long-term research with a large sample size, multiple sectors and ethnic groups has not been conducted.

Autophagy is an evolutionarily conserved process during which cellular materials are delivered to lysosomes for degradation, leading to the breakdown and eventual turnover of the resulting macromolecules [15]. Autophagy has a wide variety of physiological and pathophysiological functions, and aberrations in autophagy have been detected in many diseases [16]. Autophagy is a double-edged sword to tumor cells and the tumor itself [17]. When the autophagy of tumor cells is abnormal, autophagy facilitates the proliferation and survival of the tumor cells. However, moderate autophagy has an anti-tumor effect and acts as an inhibitor of tumors [18, 19]. Recent study demonstrated that the disorder of autophagy is closely related to the formation and development of some malignant tumors [20]. In mammals, AMPK, AMP-activated protein kinase, plays a key role in regulating cellular energy balance and helps restore homeostasis [21]. AMPK protein exists in the form of a heterotrimeric complex, which consists of an α-catalytic subunit, a β-regulatory subunit, and a γ-regulatory subunit. Humans and rodents express two subtypes of α-subunits and β-subunits (α1, α2; β1, β2) and three subtypes of γ-subunits (γ1, γ2, γ3) from different genes. It has been reported that the progression of cancer and resistance to chemotherapeutic drugs is always related to the loss of AMPK pathway in various types of cancers [22–24], and that AMPK activation can promote the activation of downstream autophagy [25, 26].

This study found that metformin could effectively inhibit the growth of AR-negative prostate cancer cells through blocking cell cycle keeping cells at G0/G1 phase. Up-regulated expressions of p-AMPK and LC3II indicated the activation of AMPK was involved in the inhibitory effect of metformin on prostate cancer through regulating autophagy. Importantly, the results obtained after the administration of AMPK siRNA or 3-MA further indicated that the AMPK/autophagy pathway might be the mechanism of metformin in treatment of prostate cancer. Moreover, we also explored the inhibitory effect of metformin on prostate cancer by performing in vivo tumor formation experiment. Our findings supported metformin as an alternative to the inhibition of the growth of AR-negative prostate cancer.

## Methods

### Chemicals

Metformin was purchased from Sigma-Aldrich (St. Louis, MO, USA). Fetal calf serum (FBS) and Dulbecco’s Modified Eagle’s Medium were purchased from HyClone (Logan, UT, USA). The autophagy inhibitor, 3-Methyladenine, was ordered from Selleck, USA. The small interfering RNA of AMPK (sc-45312) and normal control (sc-37007) came from Santa Cruz (CA, USA). BeyoClick™ EdU Cell Proliferation Kit with Alexa Fluor 555 (C0075s) was obtained from Beyotime Biotechnogy (Shanghai, China). The primary antibody of LC3B (L7543) for Western blot was obtained from Sigma-Aldrich (St. Louis, MO, USA). Other primary antibodies of cyclinD1 (AF0126), PCNA (AF1363), ERK (AF1051), p-ERK (AF5818), p62/SQSTM1 (AF5312), AMPKα (AF6195), and p-AMPK (Thr172) (AF5908) were ordered from Beyotime Biotechnology (Shanghai, China). The primary anti-LC3B antibody with Alexa Flour 647 fluorescence for immunofluorescence was from Abcam (Cambridge, MA, USA). The antibodies of LC3B (GB13431) and Ki67 (GB111499) for immunohistochemistry were attained from Servicebio (Wuhan, China). All the antibodies were used at a ratio of 1:1000 in Western blot assays, and p-AMPK (1:50), LC3B (1:300) and Ki-67 (1:600) were used in Immunohistochemistry assays.

### Cell Culture and transfection

DU145 and PC3 cells were commercially ordered from American Type Culture Collection (ATCC, Shanghai). These cells were cultured in Dulbecco’s Modified Eagle’s Medium supplemented with 10% fetal bovine serum and 1% penicillin–streptomycin (HyClone, Logan, UT, USA). The cell incubator was maintained at 37 °C with 95% air and 5% CO_2_ under the condition of humidified atmosphere.

To transfect DU145 and PC3 cells, small interfering RNA for AMPK-α1 and AMPK-α2 was used with Lipofectamine 3000 Transfection Reagent (Invitrogen, Carlsbad, CA, USA), according to the instructions. Metformin at a concentration of 20 mM was added into the culture media after transfection for 24 h (h). Then the culture media was replaced with Dulbecco’s Modified Eagle’s Medium for another 24 h culture.

### Cell viability, Colony formation experiment, cell proliferation assay, scratch assay, and cell invasion assay

DU145 and PC3 cells were seeded into a 96-well plate (3 × 10^3^ cells/well), and treated with different concentrations of metformin for 24, 48 or 72 h after cell attachment. To assess cell viability, a Cell Counting Kit (CCK)-8 (Dojindo, Kumamoto, Japan) assay was carried out. Briefly, 10 μL CCK solution was added to each well and incubated the culture plate of an incubator for 2 h. The absorbance at 450 nm was measured using a microplate reader (Thermo Fisher Scientific, Waltham, MA, USA), and the control cell viability was defined as 100%. For colony formation experiment, 300 DU145 or PC3 cells were seeded into 6-well plates, respectively. After 24 h, the cells were treated with metformin at a concentration of 0, 5, 10 or 20 mM. After 7 days, the cells were dyed by 0.2% crystal violet (Sigma-Aldrich, St. Louis, MO, USA) staining for observing colony formation. For EdU assay, 1 × 10^5^ cells were seeded onto a 6-well plate and processed with metformin for 24 h, followed by the addition of EdU. Then the cells were performed with click reaction and incubated with Hoechst 33,342. At last, the cell images were captured under a Nikon Eclipse E 400 microscope (Nikon, Fukok, Japan) [27]. The proportion of EdU positive cells, namely proliferating cells, was the ratio of Azide 555 cells (red) and the sum of Azide 555 (red) and Hoechst (blue) cells. In the scratch assay, the cells were seeded into 6-well plates till 90% confluence. A scratch was made on the top center of the accumulated cells using a pipette tip. And the medium was replaced with pure DMEM with 0–2% FBS. Cell migration at 0 and 48 h after scratching was examined and recorded with a microscope (Olympus, Japan). We used Image J to measure the length between the two borders of the scratch for random 20 places to calculate the migration rate. For cell invasion assay, 1 × 10^5^ cells were counted and seeded into the 8 μm transwell inserts (Corning, New York, USA) after the inserts had been coated with 5 mg/ml matrigel (BD Biosciences, California, USA). Pure DMEM with 20% FBS and pure DMEM without FBS was respectively used in the lower and upper chamber. The cells invaded into the lower chamber were fixed with paraformaldehyde and stained with crystal violet after 24 h. Finally, the cell images were captured and counted using Nikon Eclipse E 400 microscope (Nikon, Fukok, Japan) [28]. We used Image J to measure the cell number of three different horizons.

### Cell cycle analysis

DU145 and PC3 cells were seeded into 6-well plates at a density of 1 × 10^6^ cells per well treated with different concentrations of metformin for 48 h. The cells were trypsinized and collected before centrifugation (800 g, 5 min). Moreover, the cell pellets were washed with phosphate-buffered saline (PBS) twice. 300 µL PBS was used to resuspend the final cell pellets, followed by fixation with 700 µL precooled 70% alcohol at 4℃ overnight. The cells were incubated with 2.5 μL Rnase and 25 μL propyl iodide (PI) (Dojindo, Kumamoto, Japan) in 0.5 ml assay in an incubator at 37℃ for 30 min (min) at the second day. Cell cycle distribution was analyzed by flow cytometry (Thermo Fisher Scientific, Waltham, MA, USA) [29].

### Western blot analysis

After properly processing the cells, the cells were placed on ice before adding RIPA buffer (50 mM Tris, pH 7.4), 150 mM NaCl, 1% Triton X-100, 1% sodium deoxycholate, 0.1% SDS, sodium orthovanadate, sodium fluoride (EDTA, leupeptin) with protease inhibitor (100 mM) at a proportion of 99:1. The protein lysates were quantified by BCA method, separated by 5%-15% sodium dodecyl sulfate–polyacrylamide gel electrophoresis, and transferred to a polyvinylidene difluoride membrane (Abcam, Cambridge, MA, USA). Subsequently, the membrane was sealed with 5% non-fat milk in Tris buffer saline for 90 min at room temperature. The membrane was then washed and incubated overnight at 4 ℃ with an appropriate primary antibody at 1:1000. The next day, the membrane was washed and incubated with horseradish peroxidase-coupling secondary antibody in Tris buffer saline at room temperature for 1 h. At last, the membrane was developed with BeyoECL Plus developer (Beyotime, Shanghai, China) using the Bio-Rad Molecular Imager FX.

### In vivo experiments

The research complies with the commonly accepted principle of “reduction, refinement, replacement” (3Rs). 8 male BALB/c nude mice aged 4 to 6 weeks old were purchased from Shanghai Laboratory Animal Center (Shanghai, China). After proper adaptation, 2 million DU145 cells were subcutaneously seeded into anterior armpit (a. ap) of the mice. About 2 weeks later, the mice were randomly assigned to two groups, one group of mice were daily orally administrated with 200 μL PBS, while another group of mice were orally administrated with 200 μL metformin (250 mg/kg/day). The body weight and tumor size were recorded every three days, and the tumor size was calculated with the formula of 0.5 × (longest diameter) × (shortest diameter)^2^. 21 days after the treatment, the blood of mice was collected for biochemical detection and tumor tissues were collected to be frozen in liquid nitrogen. All the procedures in these experiments were approved by the Animal Protection and Use Committee of Soochow University.

### Immunohistochemistry analysis

A small section of each tumor tissue was cut for paraffin-embedding, followed by immunohistochemical staining. The sections were stained with antibodies of p-AMPK (1:50), LC3B (1:300), and Ki-67 (1:600). Ki67 was expressed nuclear that we used positive cell count analysis. LC3B and pAMPK were expressed cytoplasmic so that we used areal density analysis. For pAMPK and LC3B: Measure the cumulative optical density (IOD) of each slice with the pixel area (pixel) as the standard unit; and the corresponding tissue area (Area); and calculate the area density (Areal Density) = IOD/Area. For Ki67: Measure the number of positive cells in the 3 fields of view of each slice; and the corresponding total cell number, and calculate the positive rate (%) = positive cell number/total cell number*100. Here, we used three tumors for every group. And we used Upright Metallurgical Microscope (model: Eclipse Ci-L microscope, Nikon, Japan) for IHC image-capturing. The software for IHC analysis was Image-Pro Plus 6.0 from Media Cybemetics (USA).

### Statistical analysis

Statistical results were processed with SPSS 16.0. The data were shown as mean ± standard deviation from at least triplicate experiments. Student’s t-test or One-way analysis of variance was used to compare two or more groups. *p < 0.05, **p < 0.01, and ***p < 0.001 were considered as statistically significant.

## Results

### Metformin suppressed the growth, migration and invasion of AR-negative prostate cancer cell lines

**Fig. 1:**
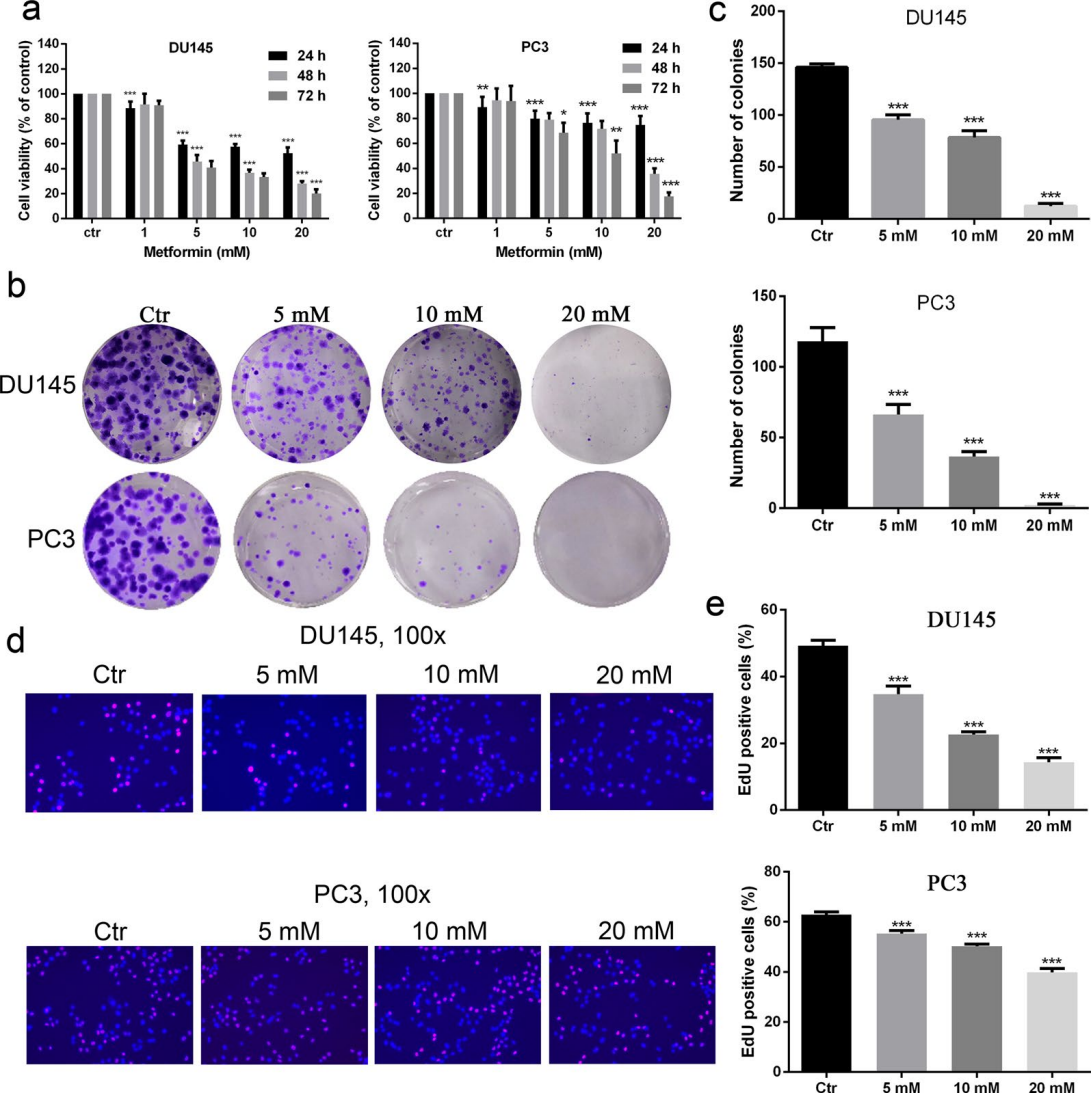
Metformin played an inhibitory role in the growth and migration of human prostate cancer cells. **a** Cell viability was determined by CCK-8 test after DU145 and PC3 cells had been treated with metformin for 24, 48, and 72 h. **b**, **c** Clone ability of DU145 and PC3 cells was detected by colony formation assay with metformin treatment. **d**, **e** Cell proliferation was examined by EdU assay. Data are presented as the mean ± SD. *p < 0.05; **p < 0.01; ***p < 0.001 compared with the control group

**Fig. 2:**
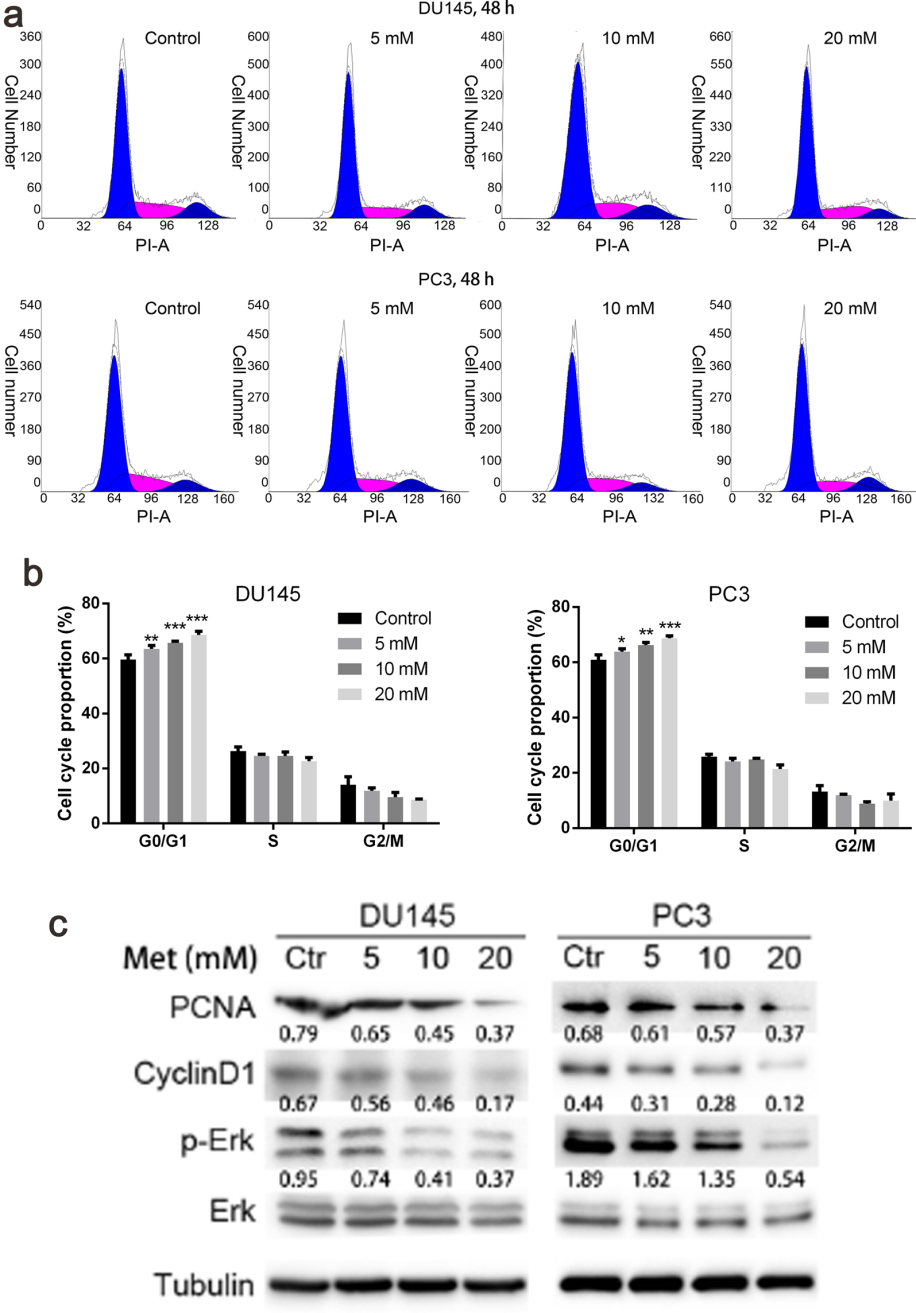
Metformin blocked cell cycle at G0/G1 phase. **a**, **b** Cell cycle distribution of metformin-treated cells was detected by flow cytometry. **c** The protein expression associated with the cell cycle was determined with Western blot analysis. The relative cyclin D1 and PCNA levels were normalized to that of α-Tubulin. p-ERK/ERK is shown. Numbers under the bands are the relative expression values to that of α-Tubulin, except for p-ERK. Data are presented as the mean ± SD. *p < 0.05; **p < 0.01; ***p < 0.001 compared with the control group. Ctr, Control; Met, Metformin

**Fig. 3:**
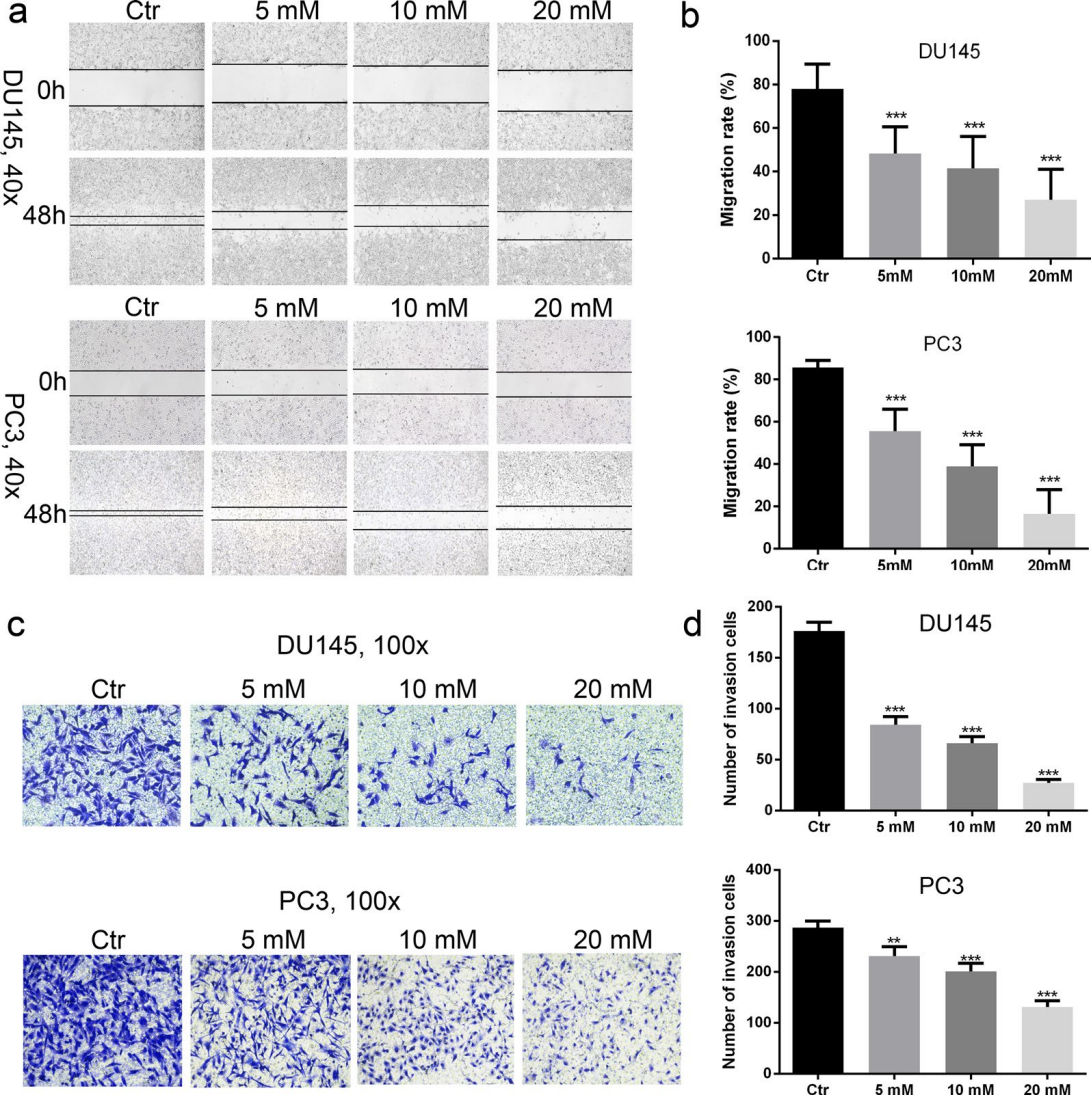
Metformin reduced the migration and invasion of AR-negative prostate cancer cells. **a**, **b** The scratch assay showed reduced migration ability of prostate cancer cells. **c**, **d** The invasion of prostate cancer cells was assessed with Transwell assay. **p < 0.01; ***p < 0.001 compared with the control group

To examine the anti-prostate cancer effect of metformin, cell viability was measured by CCK8 assay. The results showed the cell viability was gradually reducing with increased metformin concentration and prolonged time (Fig. 1a). The data from colony formation experiment demonstrated that colony formation was decreased as the concentration of metformin increased (Fig. 1b, c). Meanwhile, from cell proliferation assay, we observed that metformin inhibited cell proliferation as the concentration increased (Fig. 1d, e). Flow cytometry results demonstrated that metformin promoted the block of the cell cycle at G0/G1 phase (Fig. 2a, b). In Western blot, the expression levels of PCNA, p-Erk/Erk, and cyclin D1 were found to be down-regulated. These results indicated that the ERK activation might be involved in the regulation of metformin on prostate cancer (Fig. 2c). Moreover, the cell migration and invasion number were reduced with the increase of the concentration of metformin (Fig. 3a–d).

### Metformin promoted autophagy of prostate cancer cells

**Fig. 4:**
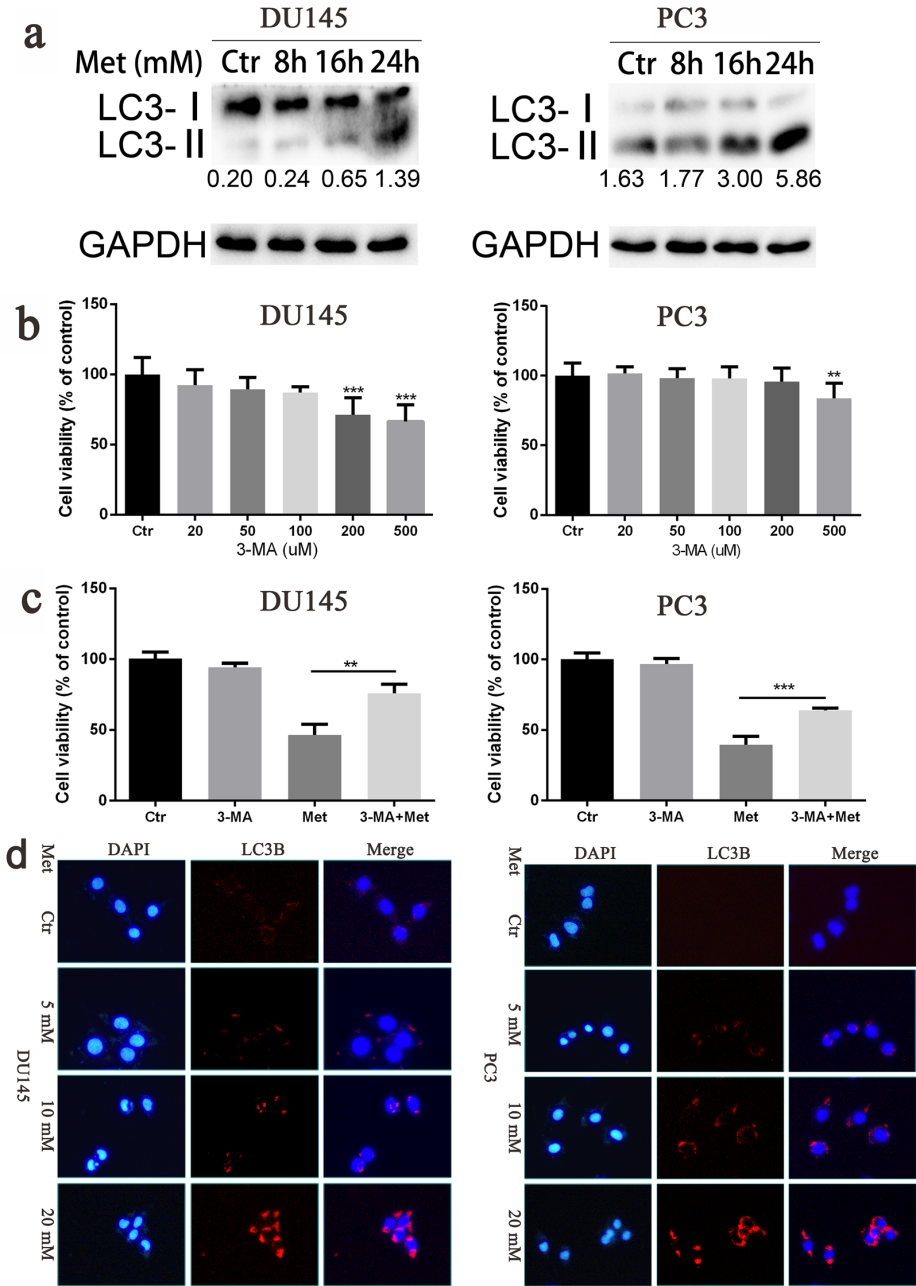
Metformin promoted autophagy of prostate cancer cells. **a** The expressions of LC3I and LC3II were detected by Western blot after treatment with metformin for 0 h, 6 h, 12 h, and 24 h for DU145 and PC3 cells. **b** The cells were treated with the autophagic inhibitor, 3-MA, for 24 h, and the viability of cells were tested by CCK8 assay. **c** The cells were treated with 3-MA at 50 μM for 2 h before treated with metformin at 20 mM, and the viability of cells were tested by CCK8 assay. **d** The cells were intervened with metformin at different concentrations for 24 h and assessed by immunofluorescence assay with an anti‐LC3 antibody. Numbers under the bands (LC3‐II for LC3) are the relative expression values to that of LC3I. Data are presented as the mean ± SD. **p < 0.01; ***p < 0.001 compared with the control group. Ctr, Control; Met, Metformin

Whether metformin could promote autophagy of prostate cancer cells was investigated. DU145 and PC3 cells were respectively treated with metformin for 6, 12, and 24 h at a concentration of 20 mM. Western blot results showed that the expression of LC3II, an indicator of autophagy, was up-regulated (Fig. 4a). To verify the mechanism through which metformin suppressed the proliferation and growth of prostate cancer cells, the cells were treated with 3-MA (an autophagy inhibitor) in advance for 2 h. We found that 3-MA at a concentration of 50 µM did not have significant toxic effect on the prostate cancer cells (Fig. 4b). Further results also showed that 3-MA could reduce the inhibitory effect of metformin (Fig. 4c). In the immunofluorescence assay, the formation of red fluorescent protein (Alexa Flour 647)-marked LC3‐positive autophagosome was getting more with the metformin concentration increasing, which indicated the autophagic process (Fig. 4d).

### Metformin activated AMPK/autophagy signaling pathway in prostate cancer cells

**Fig. 5:**
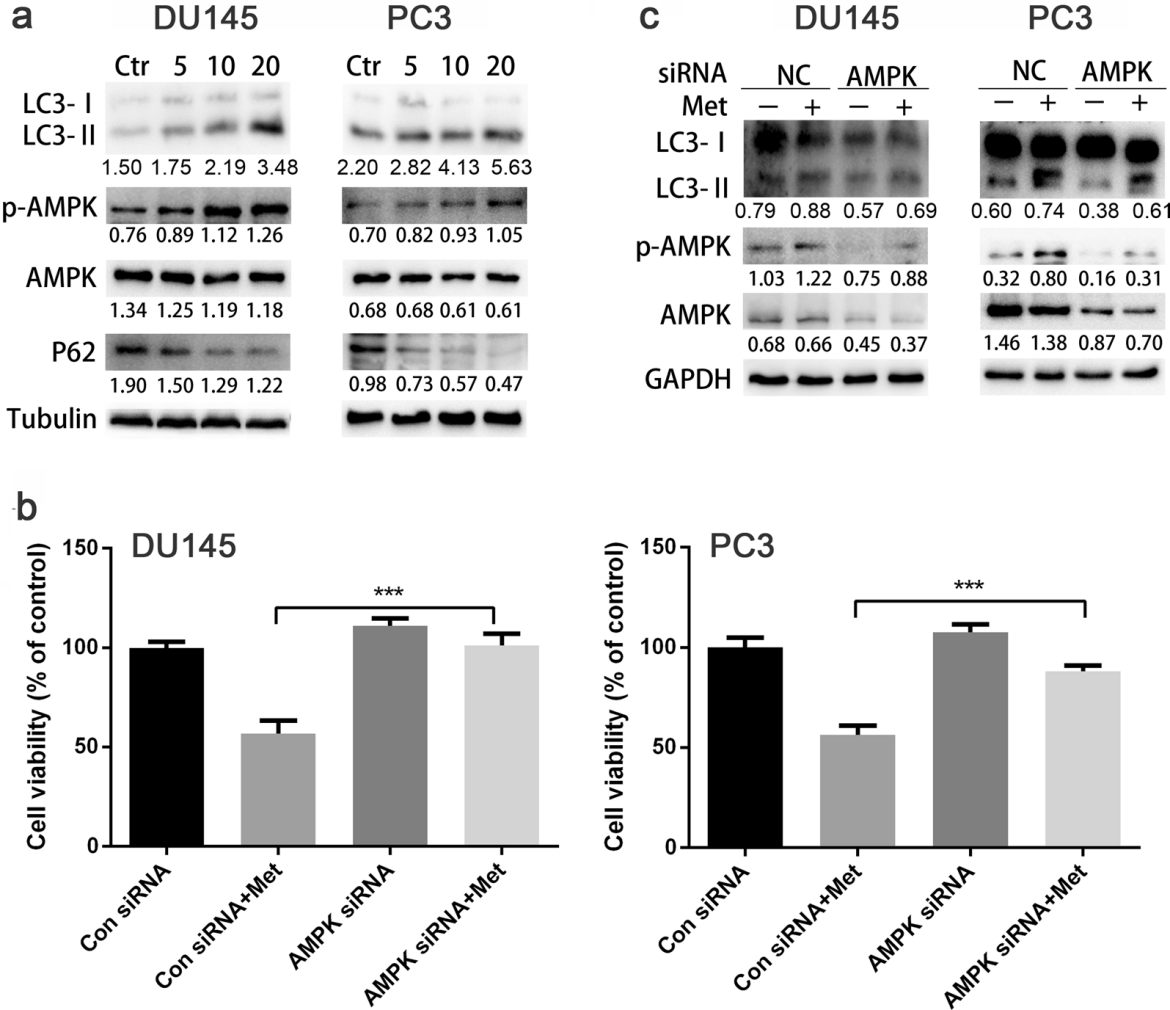
Metformin activated AMPK/autophagy signaling pathway in prostate cancer cells. **a** DU145 and PC3 cells were treated with metformin for 24 h and the expressions of p62/SQSTM1, AMPK, p-AMPK, LC3‐I and LC3‐II levels were analyzed by Western blot. **b**, **c** CCK8 assay and Western blot analysis were conducted after the cells had been treated with AMPK siRNA for 24 h first and then metformin for another 24 h. Numbers under the bands (LC3‐II for LC3) are the relative expression values to that of LC3I. Data are presented as the mean ± SD. ***p < 0.001 compared with the control group. Met, Metformin

Previous studies showed that metformin can activate AMPK, which is a key molecule mainly involved in cell growth, proliferation and autophagy. After treating DU145 and PC3 cells with metformin (5 mM, 10 mM, 20 mM) for 24 h, we observed that the phosphorylation level of AMPK (p-AMPK) and LC3II were up-regulated in DU145 and PC3 cells, while expression level of p62/SQSTM1 was down-regulated (Fig. 5a). To verify the role of AMPK in regulating the expressions of autophagy-associated proteins, we treated DU145 and PC3 cells with AMPK siRNA before adding 20 mM metformin. The results showed metformin could attenuate the cell growth promoted by AMPK siRNA (Fig. 5b, c). This showed with the reduction of AMPK, which is responsible for autophagy, autophagy was also reduced and cell growth was increased.

### Metformin resists prostate cancer in vivo

**Fig. 6:**
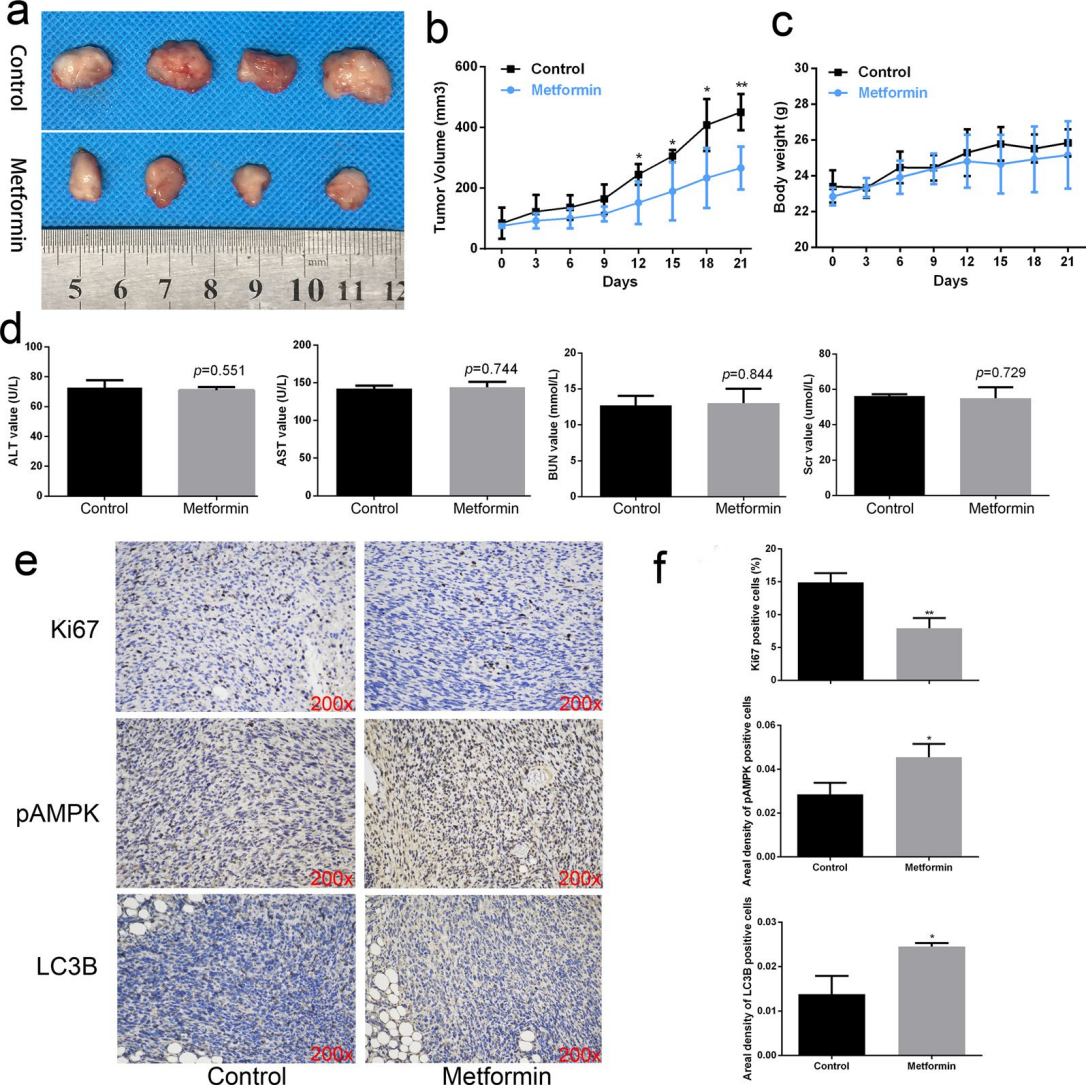
Metformin resisted prostate cancer growth in vivo. **a** Photos of tumors collected from the mice. DU145 cells were injected into anterior armpit of BALB/c male nude mice (n = 4 for each group). Then the mice were treated with metformin or PBS by daily oral administration. **b**, **c** Tumor volume and body weight of mice were recorded every 3 days. **d** The serum levels of ALT, AST, BUN, and Scr in mice from each group were detected. **e**, **f** Immunohistochemical analysis of p-AMPK, LC3B, and Ki67 for the two groups. Data are presented as the mean ± SD. *p < 0.05; **p < 0.01; ***p < 0.001 compared with the control group

Finally, we conducted animal experiments to explore the inhibitory effect of metformin on prostate cancer in vivo. Oral administration of metformin or phosphate buffer saline was adopted after subcutaneous formation of tumor using DU145 cells. We observed that the tumor of metformin group grew slower, and that the tumor size was significantly reduced in the metformin group compared with the normal control group (Fig. 6a, b). There was no significant difference in body weight between the two groups during the whole experimental process (Fig. 6c) or in biochemical related indicators (AST, ALT, BUN or Scr) (Fig. 6d). After oral administration for 21 days, we harvested the tumor tissues for immunohistochemical staining, the results of which demonstrated the expression of p-AMPK and LC3B was up-regulated and that of Ki-67 level was down-regulated in metformin group compared with normal control group (Fig. 6e, f).

## Discussion

The incidence of prostate cancer is increasing [1]. Though current treatments could help improve overall survival, we still lack an effective therapy against the cancer [30]. Metformin is a commonly used clinical drug for diabetes, and studies have increasingly shown that metformin could also prevent the occurrence of some cancers such as colorectal cancer and the prognosis of non-diabetic cancer patients, including breast cancer, thyroid cancer, and lung cancer. Moreover, combination therapy shows great potential to be a novel therapeutic strategy with significant results [31, 32]. In the present study, we found that metformin exhibited an anti-prostate cancer activity through regulating autophagy in vitro and in vivo models. In the animal study, metformin could greatly inhibit the tumor growth. Crucially, we found that AMPK siRNA suppressed autophagy, and the autophagy inhibitor, 3-MA, increased the viability of prostate cancer cells. These results suggested that AMPK/autophagy might be involved in the mechanism of metformin treatment of prostate cancer.

Metformin, a commonly used anti-diabetic drug, has been widely studied for its promising effects on inhibiting tumorigenesis and cancer development [33, 34]. Our research showed that metformin suppressed the growth of prostate cancer. Metformin blocked cell cycle at G0/G1 phase, which was in accordance with some previous studies, as metformin has also been found to be able to induce cell cycle block at G0/G1 phase in lymphoma and breast cancer [35–37]. Although metformin could suppress the growth of prostate cancer, single application of it might not be as effective as when its synergy is used with drugs in treating prostate cancer [38, 39]. Moreover, some studies also suggested the effectiveness of combined use of metformin with other promising drugs for treating prostate cancer. For osseous metastatic castration-resistant prostate cancer cells, the use of simvastatin in combination with metformin could induce G1-phase cell cycle arrest via activation of AMPK, promote autophagy [40], and inhibit the growth of LNCaP cell through activating AMPK and AKT [41]. Currently, the mechanism about the inhibitory effect of metformin on prostate cancer has not been studied.

**Fig. 7:**
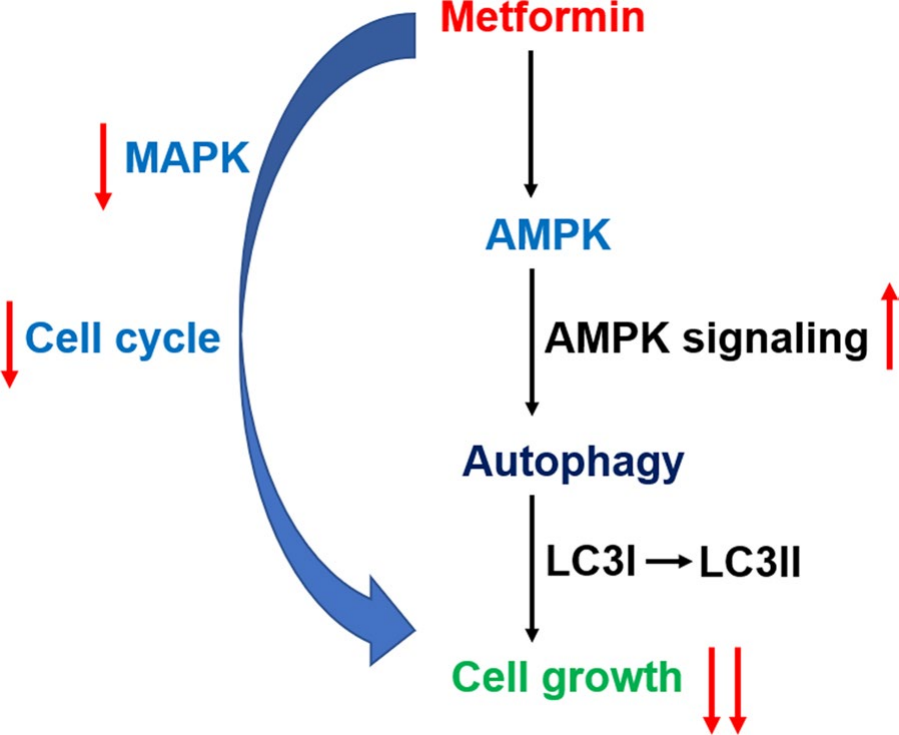
A simple schema showed how metformin affected the growth of AR-negative prostate cancer cells

Many cell-autonomous activities of metformin have been reported, primarily involving activation of AMPK pathway in breast cancer [42], inhibition of AKT-mTORC1 pathway in myeloma [43], suppression of cell cycle progression in esophageal squamous cell carcinomas [44], regulation of MAPK in lung cancer [45], SP1 transcription factor regulation in pancreatic tumor cells [42] and the unfolded protein response (UPR) in prostate cancer [46]. In our research, we discovered that metformin could reduce the ratio of p-ERK/ERK in the MAPK pathway. Cell cycle distribution results and low-expressed PCNA and cyclin D1 all indicated the block of cell cycle. AMPK, a major metabolic energy sensor, could cause autophagy [47]. Here we found that AMPK might mediate metformin-induced autophagy in prostate cancer, because we observed that knocking out AMPK promoted metformin-induced autophagy (see Fig. 7). The use of 3-MA attenuated the promotion of autophagy resulted from metformin, providing evidence for the involvement of autophagy pathway in the mechanism. At the same time, we also discovered that oral administration of metformin at a dose of 250 mg/kg produced no side effects for treating prosate cancer in vivo. In most cases, the doses of metformin used in preclinical studies in vitro and in vivo may resulted in a maximum serum level of metformin that are 10–100 times higher than those used in human clinical trials [48]. Therefore, the optional dose of metformin for clinical use should be carefully determined.

In our research, we showed that metformin could inhibit the growth of AR-negative prostate cancer cells in vitro and tumors in vivo. Clinical treatment should determine the optimal dose of metformin for patients with the cancer. Moreover, whether metformin could be used in combination with radical prostatectomy, endocrine therapy, and external radiation therapy to improve the recurrence of some prostate cancer cases were still unknown. Therefore, more comprehensive studies are required before direct application of metformin in the treatment of AR-negative prostate cancer.

## Conclusion

We found that metformin has an inhibitory role in treating AR-negative prostate cancer by blocking cell cycle and inducing autophagy via AMPK/autophagy pathway, showing a strong potential to be clinically used to treat AR-negative prostate cancer.

## Data Availability

All data generated or analyzed during this study are included in this published article.

## Abbreviations

CRPC: Castrate-resistant prostate cancer
FBS: Fetal calf serum
PBS: Phosphate‐buffered saline
PI: Propyl iodide
Met: Metformin
